# 3,3′-Dichloro­biphenyl-4,4′-diaminium sulfate

**DOI:** 10.1107/S1600536811027905

**Published:** 2011-07-16

**Authors:** Hui-Fen Qian, Wei Huang

**Affiliations:** a College of Sciences, Nanjing University of Technology, Nanjing 210009, People’s Republic of China; b State Key Laboratory of Coordination Chemistry, Nanjing National Laboratory of Microstructures, School of Chemistry and Chemical Engineering, Nanjing University, Nanjing 210093, People’s Republic of China

## Abstract

In the title compound, C_12_H_12_Cl_2_N_2_^2+^·SO_4_^2−^, the two rings are not coplanar [dihedral angle = 48.7 (2)°]. In the crystal, multiple N—H⋯O hydrogen-bond inter­actions are found between the ammonium and sulfate groups.

## Related literature

For related compounds, see: Chawdhury *et al.* (1968 ▶); Chu *et al.* (2007 ▶); Dobrzycki & Wozniak (2007 ▶); You *et al.* (2009 ▶). 
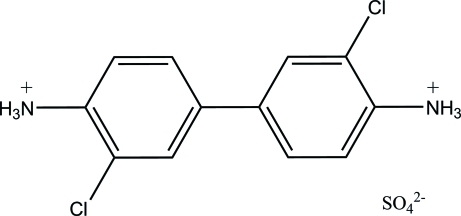


## Experimental

### 

#### Crystal data

C_12_H_12_Cl_2_N_2_^2+^·SO_4_^2−^
*M* *_r_* = 351.20
Triclinic, 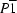

*a* = 6.5475 (11) Å
*b* = 7.9353 (13) Å
*c* = 13.363 (2) Å
α = 82.300 (2)°
β = 81.309 (3)°
γ = 88.765 (2)°
*V* = 680.12 (19) Å^3^
*Z* = 2
Mo *K*α radiation
μ = 0.65 mm^−1^
*T* = 291 K
0.12 × 0.12 × 0.10 mm

#### Data collection

Bruker 1K CCD area-detector diffractometer
Absorption correction: multi-scan (*SADABS*; Bruker, 2000 ▶) *T*_min_ = 0.926, *T*_max_ = 0.939
3484 measured reflections
2369 independent reflections
1825 reflections with *I* > 2σ(*I*)
*R*_int_ = 0.030

#### Refinement

*R*[*F* ^2^ > 2σ(*F* ^2^)] = 0.036
*wR*(*F* ^2^) = 0.091
*S* = 1.00
2369 reflections
192 parameters
H-atom parameters constrained
Δρ_max_ = 0.25 e Å^−3^
Δρ_min_ = −0.38 e Å^−3^

### 

Data collection: *SMART* (Bruker, 2000 ▶); cell refinement: *SAINT* (Bruker, 2000 ▶); data reduction: *SAINT*; program(s) used to solve structure: *SHELXTL* (Sheldrick, 2008 ▶); program(s) used to refine structure: *SHELXTL*; molecular graphics: *SHELXTL*; software used to prepare material for publication: *SHELXTL*.

## Supplementary Material

Crystal structure: contains datablock(s) global, I. DOI: 10.1107/S1600536811027905/ff2021sup1.cif

Structure factors: contains datablock(s) I. DOI: 10.1107/S1600536811027905/ff2021Isup2.hkl

Supplementary material file. DOI: 10.1107/S1600536811027905/ff2021Isup3.cml

Additional supplementary materials:  crystallographic information; 3D view; checkCIF report

## Figures and Tables

**Table 1:** Hydrogen-bond geometry (Å, °)

*D*—H⋯*A*	*D*—H	H⋯*A*	*D*⋯*A*	*D*—H⋯*A*
N1—H1*A*⋯O3^i^	0.89	1.78	2.668 (3)	173
N1—H1*B*⋯O2	0.89	2.10	2.874 (3)	144
N1—H1*C*⋯O4^ii^	0.89	1.90	2.775 (3)	167
N2—H2*A*⋯O4^iii^	0.89	1.90	2.781 (3)	172
N2—H2*B*⋯O2^iv^	0.89	1.99	2.865 (3)	166
N2—H2*C*⋯O3^v^	0.89	2.06	2.938 (3)	168

Symmetry codes: (i) 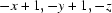; (ii) 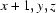; (iii) 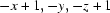; (iv) 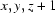; (v) 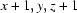.

**Fig. 1.**
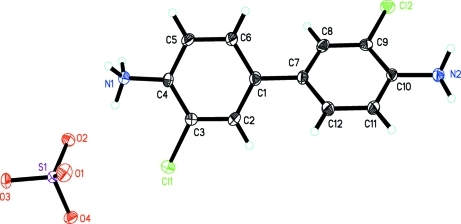
An ORTEP drawing of the title compound with the atom-numbering scheme. Displacement ellipsoids are drawn at the 30% probability level.

## supplementary crystallographic information

### Comment

There have been two single-crystal structural investigations on 4,4'-diamino-3,3'-dichlorobiphenyl, namely 4,4'-diamino-3,3'-dichlorobiphenyl (Chawdhury *et al.*, 1968) and 4,4'-Diammonio-3,3'-dichlorobiphenyl dichloride (Dobrzycki & Wozniak, 2007). We have previously reported the single-crystal structures of 2-aminobenzimidazolium hydrogen sulfate (You *et al.*, 2009) and (1*R*,3*S*)-1,2,2-trimethylcyclopentane-1,3-diammonium sulfate (Chu *et al.*, 2007). In this work, we describe the single-crystal structure of a sulfate salt of 4,4'-diamino-3,3'-dichlorobiphenyl.

The atom-numbering scheme of the title salt is shown in Fig. 1. The two phenyl rings are not coplanar with a dihedral angle of 48.7 (2)°. In the crystal packing, multiple N—H···O hydrogen-bond interactions are found between the ammonio and sulfate groups.

### Experimental

The treatment of 4,4'-diamino-3,3'-dichlorobiphenyl dissolved in methanol with an excess of sulfuric acid yields the title compound. Single crystals suitable for X-ray diffraction measurement were obtained after 5 days' slow evaporation of the mother liquid at room temperature in air. Anal. Calcd. For C_12_H_12_N_2_Cl_2_^2+^.SO_4_^2-^: C, 41.04; H, 3.44; N, 7.98%. Found: C, 41.22; H, 3.63; N, 7.79%.

### Refinement

The non-hydrogen atoms were refined anisotropically, whereas the H atoms bonded with carbon, nitrogen and oxygen atoms were placed in geometrically idealized positions (C—H = 0.93 Å and N—H = 0.89 Å) and refined as riding atoms, with *U*_iso_(H) = 1.2*U*_eq_(C) and 1.5*U*_eq_(N).

### Crystal data

**Table:**

C_12_H_12_Cl_2_N_2_^2+^·SO_4_^2^^−^	*Z* = 2
*M**_r_* = 351.20	*F*(000) = 360
Triclinic, *P*1	*D*_x_ = 1.715 Mg m^−^^3^
Hall symbol: -P 1	Mo *K*α radiation, λ = 0.71073 Å
*a* = 6.5475 (11) Å	Cell parameters from 1332 reflections
*b* = 7.9353 (13) Å	θ = 2.8–27.6°
*c* = 13.363 (2) Å	µ = 0.65 mm^−^^1^
α = 82.300 (2)°	*T* = 291 K
β = 81.309 (3)°	Block, colourless
γ = 88.765 (2)°	0.12 × 0.12 × 0.10 mm
*V* = 680.12 (19) Å^3^	

### Data collection

**Table:**

Bruker 1K CCD area-detector diffractometer	2369 independent reflections
Radiation source: fine-focus sealed tube	1825 reflections with *I* > 2σ(*I*)
graphite	*R*_int_ = 0.030
φ and ω scans	θ_max_ = 25.0°, θ_min_ = 2.6°
Absorption correction: multi-scan (*SADABS*; Bruker, 2000)	*h* = −7→7
*T*_min_ = 0.926, *T*_max_ = 0.939	*k* = −8→9
3484 measured reflections	*l* = −8→15

### Refinement

**Table:**

Refinement on *F*^2^	Primary atom site location: structure-invariant direct methods
Least-squares matrix: full	Secondary atom site location: difference Fourier map
*R*[*F*^2^ > 2σ(*F*^2^)] = 0.036	Hydrogen site location: inferred from neighbouring sites
*wR*(*F*^2^) = 0.091	H-atom parameters constrained
*S* = 1.00	*w* = 1/[σ^2^(*F*_o_^2^) + (0.0394*P*)^2^] where *P* = (*F*_o_^2^ + 2*F*_c_^2^)/3
2369 reflections	(Δ/σ)_max_ < 0.001
192 parameters	Δρ_max_ = 0.25 e Å^−^^3^
0 restraints	Δρ_min_ = −0.38 e Å^−^^3^

### Special details

**Table:**

Experimental. The structure was solved by direct methods (Bruker, 2000) and successive difference Fourier syntheses.
Geometry. All e.s.d.'s (except the e.s.d. in the dihedral angle between two l.s. planes) are estimated using the full covariance matrix. The cell e.s.d.'s are taken into account individually in the estimation of e.s.d.'s in distances, angles and torsion angles; correlations between e.s.d.'s in cell parameters are only used when they are defined by crystal symmetry. An approximate (isotropic) treatment of cell e.s.d.'s is used for estimating e.s.d.'s involving l.s. planes.
Refinement. Refinement of *F*^2^ against ALL reflections. The weighted *R*-factor *wR* and goodness of fit *S* are based on *F*^2^, conventional *R*-factors *R* are based on *F*, with *F* set to zero for negative *F*^2^. The threshold expression of *F*^2^ > σ(*F*^2^) is used only for calculating *R*-factors(gt) *etc*. and is not relevant to the choice of reflections for refinement. *R*-factors based on *F*^2^ are statistically about twice as large as those based on *F*, and *R*- factors based on ALL data will be even larger.

### Fractional atomic coordinates and isotropic or equivalent isotropic displacement parameters (Å^2^)

**Table:**

	*x*	*y*	*z*	*U*_iso_*/*U*_eq_	
C1	0.7508 (4)	0.2990 (3)	0.4692 (2)	0.0268 (6)	
C2	0.5748 (4)	0.2423 (3)	0.4365 (2)	0.0282 (6)	
H2	0.4653	0.1965	0.4843	0.034*	
C3	0.5626 (4)	0.2540 (3)	0.3332 (2)	0.0264 (6)	
C4	0.7242 (4)	0.3252 (3)	0.2613 (2)	0.0236 (6)	
C5	0.8970 (4)	0.3847 (4)	0.2935 (2)	0.0290 (7)	
H5	1.0043	0.4346	0.2458	0.035*	
C6	0.9099 (4)	0.3697 (4)	0.3965 (2)	0.0298 (7)	
H6	1.0278	0.4079	0.4174	0.036*	
C7	0.7701 (4)	0.2740 (3)	0.5796 (2)	0.0259 (6)	
C8	0.9509 (4)	0.2014 (3)	0.6102 (2)	0.0291 (7)	
H8	1.0581	0.1721	0.5616	0.035*	
C9	0.9706 (4)	0.1731 (3)	0.7124 (2)	0.0252 (6)	
C10	0.8127 (4)	0.2160 (3)	0.7859 (2)	0.0230 (6)	
C11	0.6338 (4)	0.2909 (3)	0.7563 (2)	0.0273 (6)	
H11	0.5279	0.3217	0.8051	0.033*	
C12	0.6141 (4)	0.3193 (3)	0.6538 (2)	0.0277 (6)	
H12	0.4943	0.3697	0.6342	0.033*	
Cl1	0.35323 (11)	0.16699 (10)	0.29449 (6)	0.0399 (2)	
Cl2	1.19670 (10)	0.08523 (9)	0.74738 (6)	0.0351 (2)	
N1	0.7263 (3)	0.3316 (3)	0.15195 (16)	0.0266 (5)	
H1A	0.7437	0.4386	0.1222	0.040*	
H1B	0.6069	0.2923	0.1404	0.040*	
H1C	0.8295	0.2678	0.1263	0.040*	
N2	0.8311 (3)	0.1779 (3)	0.89363 (16)	0.0267 (5)	
H2A	0.8553	0.0673	0.9087	0.040*	
H2B	0.7141	0.2058	0.9310	0.040*	
H2C	0.9351	0.2373	0.9071	0.040*	
O1	0.2424 (3)	0.4428 (2)	0.08113 (15)	0.0381 (5)	
O2	0.4527 (3)	0.2079 (2)	0.02918 (15)	0.0335 (5)	
O3	0.2129 (3)	0.3412 (2)	−0.07783 (14)	0.0306 (5)	
O4	0.0875 (3)	0.1693 (2)	0.07938 (15)	0.0311 (5)	
S1	0.25232 (10)	0.29314 (8)	0.02901 (5)	0.02355 (19)	

### Atomic displacement parameters (Å^2^)

**Table:**

	*U*^11^	*U*^22^	*U*^33^	*U*^12^	*U*^13^	*U*^23^
C1	0.0310 (15)	0.0270 (15)	0.0227 (15)	−0.0027 (12)	−0.0041 (13)	−0.0045 (12)
C2	0.0283 (15)	0.0300 (16)	0.0234 (15)	−0.0024 (12)	0.0028 (12)	0.0000 (13)
C3	0.0234 (14)	0.0286 (16)	0.0269 (16)	−0.0015 (12)	−0.0037 (12)	−0.0029 (13)
C4	0.0298 (15)	0.0206 (14)	0.0195 (14)	0.0013 (11)	−0.0039 (12)	0.0002 (11)
C5	0.0284 (15)	0.0311 (16)	0.0257 (16)	−0.0100 (12)	0.0008 (13)	−0.0011 (13)
C6	0.0311 (15)	0.0347 (17)	0.0239 (15)	−0.0073 (13)	−0.0038 (13)	−0.0047 (13)
C7	0.0301 (15)	0.0286 (15)	0.0193 (15)	−0.0046 (12)	−0.0033 (12)	−0.0040 (12)
C8	0.0264 (15)	0.0346 (17)	0.0249 (16)	−0.0005 (12)	0.0041 (13)	−0.0077 (13)
C9	0.0232 (14)	0.0220 (14)	0.0303 (16)	−0.0028 (11)	−0.0023 (12)	−0.0042 (12)
C10	0.0261 (14)	0.0231 (14)	0.0198 (14)	−0.0046 (11)	−0.0033 (12)	−0.0019 (12)
C11	0.0249 (14)	0.0334 (16)	0.0235 (15)	0.0029 (12)	−0.0010 (12)	−0.0069 (13)
C12	0.0270 (15)	0.0300 (16)	0.0258 (16)	0.0030 (12)	−0.0065 (13)	−0.0008 (13)
Cl1	0.0288 (4)	0.0558 (5)	0.0356 (5)	−0.0112 (3)	−0.0081 (3)	−0.0029 (4)
Cl2	0.0242 (4)	0.0399 (4)	0.0404 (5)	0.0043 (3)	−0.0044 (3)	−0.0040 (4)
N1	0.0275 (12)	0.0301 (13)	0.0220 (13)	−0.0012 (10)	−0.0030 (10)	−0.0027 (11)
N2	0.0269 (12)	0.0304 (13)	0.0233 (13)	0.0014 (10)	−0.0039 (10)	−0.0057 (11)
O1	0.0500 (13)	0.0323 (12)	0.0339 (12)	−0.0032 (10)	−0.0032 (10)	−0.0142 (10)
O2	0.0250 (10)	0.0389 (12)	0.0351 (12)	0.0058 (9)	−0.0044 (9)	−0.0010 (10)
O3	0.0355 (11)	0.0356 (12)	0.0209 (10)	0.0000 (9)	−0.0091 (9)	0.0008 (9)
O4	0.0269 (10)	0.0288 (11)	0.0346 (12)	−0.0033 (8)	0.0008 (9)	0.0013 (9)
S1	0.0221 (4)	0.0265 (4)	0.0211 (4)	−0.0020 (3)	−0.0017 (3)	−0.0015 (3)

### Geometric parameters (Å, °)

**Table:**

C1—C6	1.385 (4)	C9—Cl2	1.724 (3)
C1—C2	1.396 (4)	C10—C11	1.389 (3)
C1—C7	1.486 (4)	C10—N2	1.454 (3)
C2—C3	1.386 (4)	C11—C12	1.383 (4)
C2—H2	0.9300	C11—H11	0.9300
C3—C4	1.391 (4)	C12—H12	0.9300
C3—Cl1	1.725 (3)	N1—H1A	0.8900
C4—C5	1.383 (4)	N1—H1B	0.8900
C4—N1	1.453 (3)	N1—H1C	0.8900
C5—C6	1.381 (4)	N2—H2A	0.8900
C5—H5	0.9300	N2—H2B	0.8900
C6—H6	0.9300	N2—H2C	0.8900
C7—C12	1.389 (4)	O1—S1	1.4506 (19)
C7—C8	1.398 (4)	O2—S1	1.4636 (18)
C8—C9	1.379 (4)	O3—S1	1.4871 (19)
C8—H8	0.9300	O4—S1	1.4939 (19)
C9—C10	1.384 (4)		
			
C6—C1—C2	118.6 (3)	C9—C10—C11	119.7 (2)
C6—C1—C7	121.2 (2)	C9—C10—N2	120.2 (2)
C2—C1—C7	120.1 (3)	C11—C10—N2	120.1 (2)
C3—C2—C1	120.3 (3)	C12—C11—C10	119.6 (2)
C3—C2—H2	119.8	C12—C11—H11	120.2
C1—C2—H2	119.8	C10—C11—H11	120.2
C2—C3—C4	120.2 (2)	C11—C12—C7	121.2 (2)
C2—C3—Cl1	119.3 (2)	C11—C12—H12	119.4
C4—C3—Cl1	120.3 (2)	C7—C12—H12	119.4
C5—C4—C3	119.6 (2)	C4—N1—H1A	109.5
C5—C4—N1	117.4 (2)	C4—N1—H1B	109.5
C3—C4—N1	122.8 (2)	H1A—N1—H1B	109.5
C6—C5—C4	119.8 (3)	C4—N1—H1C	109.5
C6—C5—H5	120.1	H1A—N1—H1C	109.5
C4—C5—H5	120.1	H1B—N1—H1C	109.5
C5—C6—C1	121.4 (3)	C10—N2—H2A	109.5
C5—C6—H6	119.3	C10—N2—H2B	109.5
C1—C6—H6	119.3	H2A—N2—H2B	109.5
C12—C7—C8	118.6 (2)	C10—N2—H2C	109.5
C12—C7—C1	122.3 (2)	H2A—N2—H2C	109.5
C8—C7—C1	119.1 (2)	H2B—N2—H2C	109.5
C9—C8—C7	120.2 (2)	O1—S1—O2	111.47 (11)
C9—C8—H8	119.9	O1—S1—O3	109.96 (12)
C7—C8—H8	119.9	O2—S1—O3	109.85 (11)
C8—C9—C10	120.7 (2)	O1—S1—O4	110.49 (12)
C8—C9—Cl2	118.9 (2)	O2—S1—O4	108.41 (11)
C10—C9—Cl2	120.3 (2)	O3—S1—O4	106.53 (11)

### Hydrogen-bond geometry (Å, °)

**Table:**

*D*—H···*A*	*D*—H	H···*A*	*D*···*A*	*D*—H···*A*
N1—H1A···O3^i^	0.89	1.78	2.668 (3)	173
N1—H1B···O2	0.89	2.10	2.874 (3)	144
N1—H1C···O4^ii^	0.89	1.90	2.775 (3)	167
N2—H2A···O4^iii^	0.89	1.90	2.781 (3)	172
N2—H2B···O2^iv^	0.89	1.99	2.865 (3)	166
N2—H2C···O3^v^	0.89	2.06	2.938 (3)	168

Symmetry codes: (i) −*x*+1, −*y*+1, −*z*; (ii) *x*+1, *y*, *z*; (iii) −*x*+1, −*y*, −*z*+1; (iv) *x*, *y*, *z*+1; (v) *x*+1, *y*, *z*+1.

## References

[bb1] Bruker (2000). *SMART*, *SAINT* and *SADABS* Bruker AXS Inc., Madison, Wisconsin, USA.

[bb2] Chawdhury, S. A., Hargreaves, A. & Rizvi, S. H. (1968). *Acta Cryst.* B**24**, 1633–1638.

[bb3] Chu, Z.-L., Fan, Y., Huang, W. & Liu, J.-L. (2007). *Acta Cryst.* E**63**, o4927.

[bb4] Dobrzycki, L. & Wozniak, K. (2007). *CrystEngComm*, **9**, 1029–1041.

[bb5] Sheldrick, G. M. (2008). *Acta Cryst.* A**64**, 112–122. 10.1107/S0108767307043930 18156677

[bb6] You, W., Fan, Y., Qian, H.-F., Yao, C. & Huang, W. (2009). *Acta Cryst.* E**65**, o115. 10.1107/S1600536808041706 PMC2968037 21581577

